# Supplementary figures and images for: Insights into the Mechanisms of Heat Priming and Thermotolerance in Tobacco Pollen
Source: Int J Mol Sci. 2021 Aug 8;22(16):8535. doi: 10.3390/ijms22168535 (PMC8395212; doi:10.3390/ijms22168535)

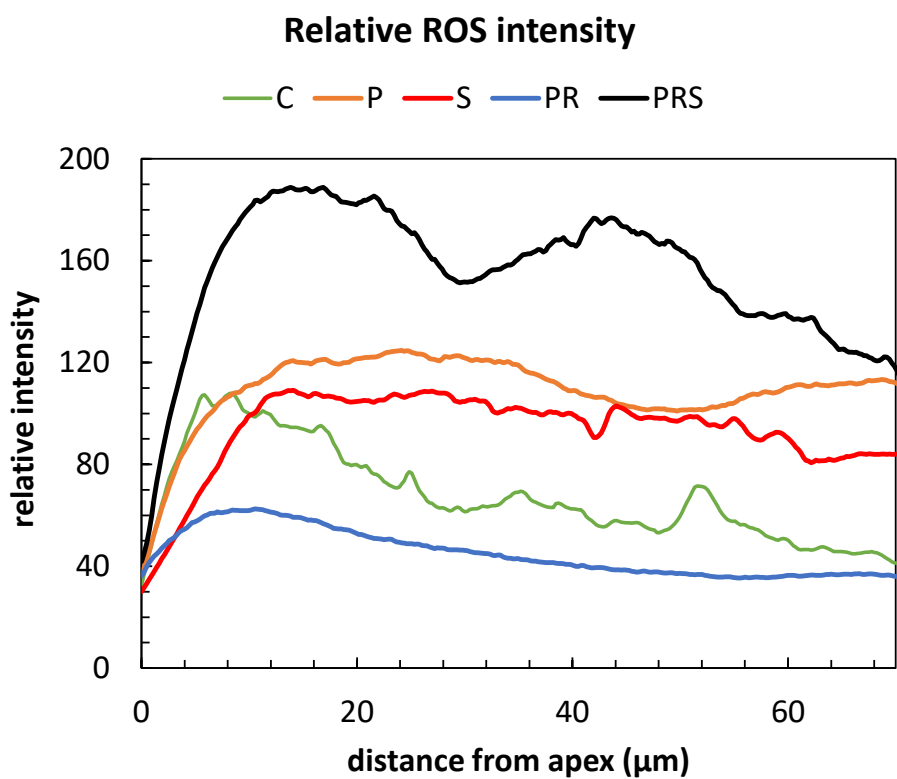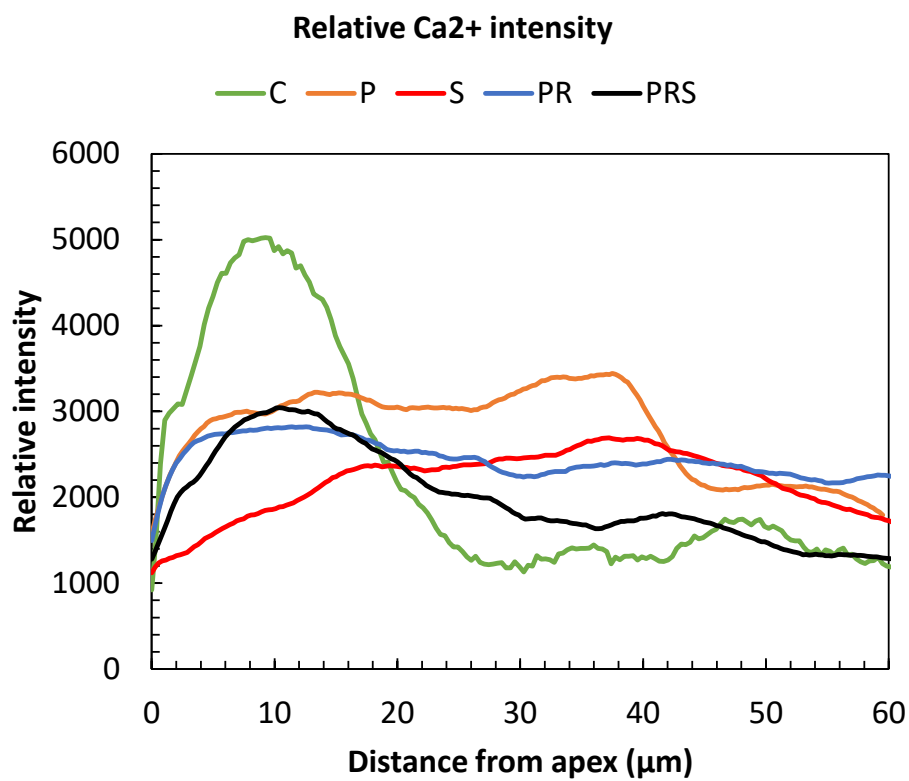

Supplement: Supplementary file 1 [file ijms-22-08535-s001.zip › ijms-1318644-supplementary.pdf]
